# Prevalence of aeroallergen sensitization in a polluted and industrialized area: a pilot study in South Africa’s Vaal Triangle

**DOI:** 10.1007/s10661-025-13718-y

**Published:** 2025-02-13

**Authors:** Dorra Gharbi, Frank Harald Neumann, Jurgens Staats, Marinda McDonald, Jo-hanné Linde, Tshiamo Mmatladi, Keneilwe Podile, Stuart Piketh, Roelof Burger, Rebecca M. Garland, Petra Bester, Pedro Humberto Lebre, Cristian Ricci

**Affiliations:** 1 Institute of Plant Sciences, University of Bern, Bern, Switzerland; 2 Oeschger Centre for Climate Change Research, University of Bern, Bern, Switzerland; 3 Unit for Environmental Sciences and Management, Faculty of Natural and Agricultural Science, North-West University, Potchefstroom, South Africa; 4 Faculty of Health Sciences, North-West University, Potchefstroom, South Africa; 5 The Allergy Clinic, Blairgowrie, Gauteng South Africa; 6 Department of Geography, Geoinformatics and Meteorology, University of Pretoria, Pretoria, South Africa; 7 Africa Unit for Transdisciplinary Health Research, North-West University, Potchefstroom, South Africa; 8 Centre for Microbial Ecology and Genomics, University of Pretoria, Pretoria, South Africa

**Keywords:** South Africa, Skin prick testing, Allergens, Pollen, Fungal, Allergic rhinitis, Public health

## Abstract

This pioneering study evaluates the prevalence of aeroallergens reactivity among atopic populations living in the Vaal Triangle Airshed Priority Area (VTAPA), South Africa. A total of 138 volunteers (51 males and 87 females), of African, colored, white, and Asian ethnicity, and with a mean (range) age of 22 (18–56) years were participating in the study. The study was conducted on the North-West University (NWU) campus in Vanderbijlpark/VTAPA. The International Study of Asthma and Allergies in Childhood questionnaire was utilized for pre-screening to identify individuals with probable allergic dispositions. Subsequently, skin prick testing was conducted using commercial aeroallergen extracts for all confirmed participants with allergy symptoms. One hundred six participants were clinically diagnosed with pollen and fungal spore allergies. The highest allergy prevalence was attributed to *Cynodon dactylon* ((L.) Pers) (Bermuda grass) (41.5%), followed by *Lolium perenne* (L.) (ryegrass), grass mix, and *Zea mays* (L.) (maize) (31.1%), respectively. Moreover, among the tree allergens, *Olea* (L.) (olive tree) was the most prevalent allergen (20; 18.8%), followed by *Platanus* (L.) (plane tree) (18; 16.9%). Among the weeds, 16 (15.1%) participants were allergic to the weed mix (*Artemisia* (*L.*) (wormwood), *Chenopodium* (Link) (goosefoot), *Salsola* (L.) (saltwort), *Plantago* (L.) (plantain), and 11 (10.3%) to *Ambrosia* (L.) (ragweed)). Regarding the fungal spores, *Alternaria* (Fr.) (9; 8.5%) followed by *Cladosporium* (Link) (5; 4.7%) had the highest skin sensitivity. In this pilot study, our findings provide insights into the prevalence of allergic responses in the study population—underlining the strong impact of allergens of exotic plants—and contribute to the existing aerobiological data in South Africa.

## Introduction

Allergic diseases such as bronchial asthma and allergic rhinitis are globally increasing (Bousquet et al., 2020). Outdoor biological particles such as airborne pollen and fungal spores are recognized as the main causes of allergic respiratory diseases globally (Grewling et al., 2023). Consequently, it is mandatory to understand the biological particle profiles, their main local vegetation sources, interaction with weather conditions, and respiratory health metrics, in a specific geographical area (Rojo et al., 2024). Monitoring aerobiological particles, particularly pollen and fungal spores, which contribute to allergies and conditions such as asthma, rhinitis, and hypersensitivity pneumonitis, is considered a valuable tool in occupational medicine (Lancia et al., 2021). Multiple studies have suggested that aerobiological outdoor measurements and clinical data must be combined in the same regions to improve allergic patients’ diagnosis and optimal treatment (Zemmer et al., 2022). The global aerobiology research community has confirmed a link between allergic symptoms and fluctuations in airborne pollen and fungal spore concentrations (Beck et al. 2021; Bonini et 2022). Additionally, a qualitative linear response relationship exists between specific local allergen exposure and symptoms of allergic diseases (Al Nesf et al., 2020; Lee et al., 2021; Laha et al., 2023). Epidemiological studies suggest a potential link between rising global allergic disease prevalence and increased air pollution (Capone et al., 2023; Schiavoni et al., 2017). Botanical studies suggest a significant effect of several pollutants combined with climatic changes on the increased expression of allergenic proteins in several pollen grains (Prodić et al., 2024; Galveias et al., 2021).

In light of the high diversity in aeroallergens within a geographical region and the key aspect of the complexity of respiratory allergic diseases, it is important to consider the appropriate skin prick test panel of inhalant allergens based on local aeroallergen exposure in clinical trial design (Anggraeni et al., 2021; Castor et al., 2024). Skin prick testing (SPT) is the most reliable diagnostic method to detect immunoglobulin (Ig) E-mediated type-I hypersensitivity reactions, particularly in allergy sufferers (Heinzerling et al., 2013). SPT is regarded as simple, safe, sensitive, inexpensive, and efficient in producing repeatable results (Şahin et al., 2020).

In contrast to the Global North, the Global South including Africa has a massive deficiency in both the primary datasets of allergy prevalence and the associated spectra of environmental exposure (Katelaris et al., 2012). Against that backdrop of respiratory diseases, the burden of allergic respiratory disease is immense. An estimated one in ten adolescents has severe asthma (overall prevalence of 13.7%); more than 30% of South Africa’s adult population suffers from allergic rhinitis (Mphahlele et al. 2023; Richards et al., 2023). Despite an asthma prevalence that is only marginally higher than the global average of ~ 10%, asthma mortality remains disproportionately high compared to economically equivalent countries, likely related to high burdens of undiagnosed disease, elevated levels of air pollution, and delayed treatment (Mortimer et al., 2022).

Furthermore, in many cases, core respiratory health metrics are insufficiently integrated with environmental air sampling, particularly aerobiological monitoring data. The blending of these intersecting exposure and respiratory health data is the starting point for developing a relevant pioneer approach around implementing health measures strategies, i.e., aeroallergen environmental exposure and sensitization prevalence in different communities in South Africa (Borstlap et al., 2014; Hoek et al., 2021; Olaniyan, 2018).

From an ecological perspective, South Africa is the third most biologically diverse country (Cadman et al., 2010) with nine biomes containing numerous vegetation units, each with its unique climate, soils, flora, and fauna (Rutherford 1994; Rutherford et al., 2006). A recent publication on the aerobiology of South Africa, presenting pollen assemblages from seven cities in different biomes provides extensive details of all biomes and matches this with a significant diversity of pollen exposures (Esterhuizen et al., 2023) and the high dominance of exotic trees linked to the country’s colonial past, e.g., oak, pine, poplar, birch, and plane trees (Gharbi et al., 2023). Moreover, a call for a better image regarding the regional dispersal of allergenic pollen and spores was initiated due to ongoing aerobiological monitoring efforts by the South African Pollen Monitoring Network in a growing number of cities in diverse biomes, such as North-West province (Neumann et al., 2024).

Although the high occurrence of aerospora (airborne pollen and fungal spores), aeroallergen skin prick testing and the interpretation of results were investigated only in the Northern Cape, where previous studies have demonstrated that allergies to grass pollen and mold spores are present in South Africa, specifically the Northern Cape (Van Rooyen et al., 2020). The prevalence of sensitization and the development of aeroallergen symptoms in different regions of South Africa remain a focus of ongoing research (Van Rooyen et al., 2020).

A research project was initiated by aerobiologists, air quality scientists, public health experts, and allergologists with the aim to focus on respiratory health impacts from allergenic pollen and fungal spores and air quality in a low-income area of South Africa within the Vaal Triangle Airshed Priority Area (VTAPA). The target area was previously declared a priority area for research due to the high levels of air pollution in the region (Muyemeki et al., 2022) and the fact that poor air quality is causing respiratory diseases (Phaswana et al., 2022). Furthermore, this study addresses the research lacuna to determine the allergenic biological sources and sensitization rate in atopic adults living in the studied area by employing a two-step approach to allergy screening and diagnosis in VTAPA. This case study offers a novel approach by combining ISAAC screening with skin prick testing, providing a comprehensive assessment of environmental allergic sensitization in a South African context.

## Material and methods

### Study area

**Fig. 1:**
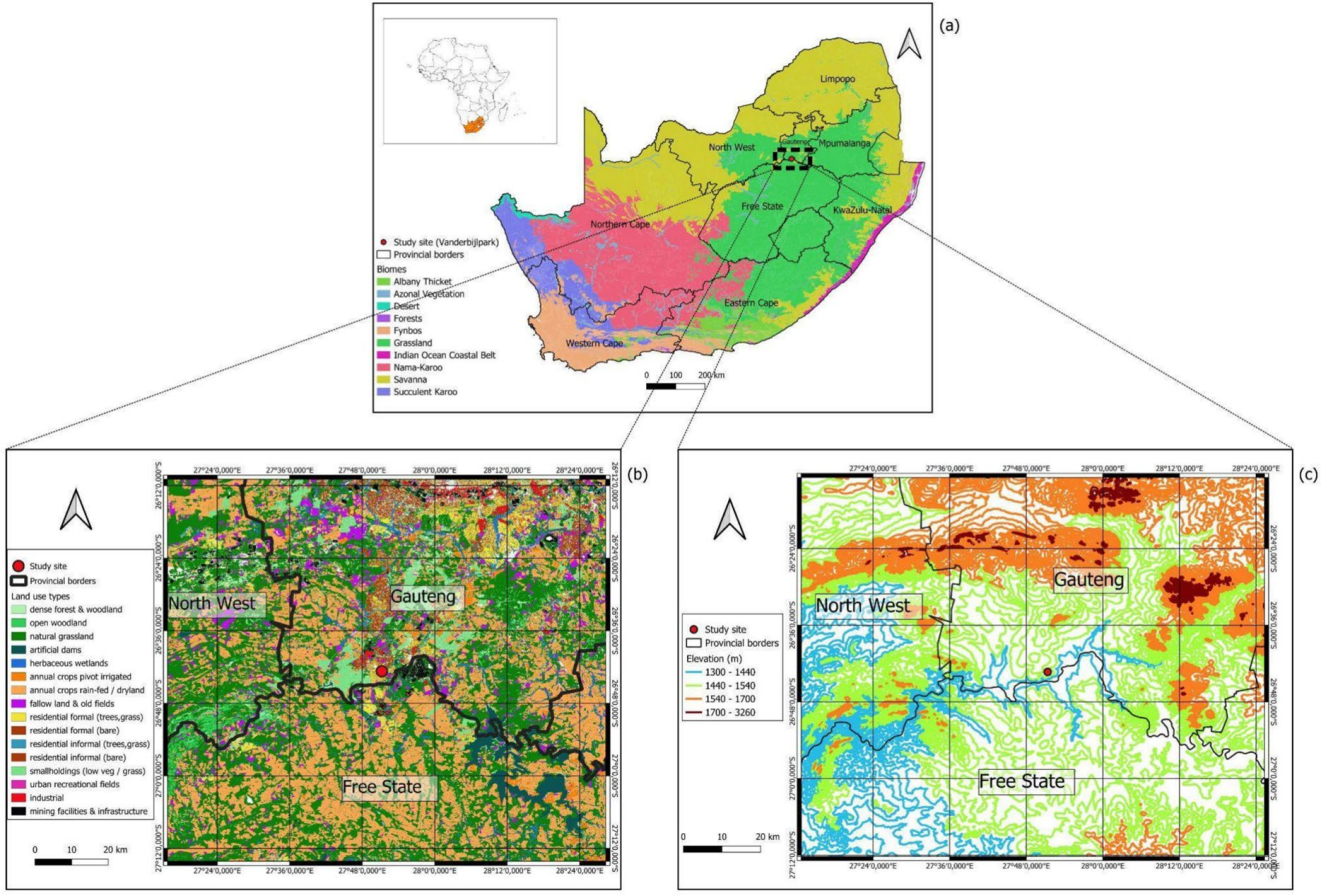
Study location. **a** Biome map (after Rutherford et al., 2006), **b** land use map, and **c** topography (designed with QGIS); the red dot represents the study area

The study site is in Vanderbijlpark (S 26° 43′ 31.0″ E 27°52′ 45.2″), a fast-growing working-class city at an altitude of c. 1500 m at the banks of the Vaal River (Fig. 1c) and surrounded by vast townships (e.g., Sharpeville), in the Gauteng Province. The study site is within the strongly industrialized VTAPA, which was declared due to poor air quality a priority area for interventions to monitor and mitigate pollution (Muyemeki et al., 2022). The high air pollution is due to a range of emissions from sectors such as industry (e.g., steel production, petrochemical factories, mining), agriculture, coal-fired power plants, transport, and household fuels (Fig. 1b) (Scorgie et al., 2003; Wright et al., 2011). The air pollution in VTAPA regularly exceeds the South African National Ambient Air Quality Standards, driven mostly by high levels of particulate matter such as in Sharpeville where annual PM2.5 concentration levels above 70 µg/m^3^ were measured (Govender & Sivakumar, 2019). Low air quality, linked to the increase of PM 2.5 and SO2, impacts public health through several ailments ranging from a decline of lung function and low-level upper respiratory irritation to severe chronic respiratory and cardiovascular diseases (Phaswana et al., 2022).

The study region experiences a summer-rainfall climate with an average annual precipitation of c. 650 mm. It has a cool-temperate climate characterized by high-temperature extremes between summer and winter, frequent frost, and significant diurnal temperature variation, particularly in autumn and spring (Rutherford et al., 2006). The region in the surroundings of Vanderbijlpark belongs to the temperate Grassland Biome, more specifically to the Soweto Highveld Grassland (GM8) which is characterized by a gently undulating landscape with dense, tufted grassland dominated by *Themeda triandra* (Forssk.) (red grass), along with other grasses like *Elionurus muticus* (Thunb.) (tall feather grass) and *Eragrostis racemosa* (Wahlenb.) (bushveld lovegrass) (Rutherford et al., 2006, revised after Desmet et al., 2024).

Common herbs are *Hermannia depressa* (Burm.f.) (trailing bushmallow), *Acalypha angustata* (Eckl. & Zeyh.) (narrow-leaved copperleaf) as well as diverse Asteraceae, e.g., *Helichrysum* (Roth.) (strawflower) spp. (Rutherford et al., 2006). Shrubs are rare and include *Anthospermum hispidulum* (Schumach.) (rough-leaved coffeeberry) and *Ziziphus zeyheriana* (J.P. Mimm.) (wild jujube), but a large part of the area except for a few regions such as in Waldrift 5 km to the north of Vanderbijlpark has been altered by cultivation, urban expansion, mining, and the construction of road infrastructure (Rutherford et al., 2006). Consequently, exotic weeds such as *Ambrosia* (L.) (ragweed) spp. from Northern America, currently spreading, especially in northern South Africa with species such as *Ambrosia artemisiifolia* (L.) (common ragweed) and *Ambrosia trifida* (L.) (giant ragweed), occur (compare Gharbi et al., 2024, Neumann et al., 2024). In Vanderbijlpark, where residential and industrial areas dominate, recreational spaces, e.g., along the Vaal River where the NWU Vanderbijlpark campus is situated, will contain exotic trees such as *Salix babylonica* (L.) (weeping willow), *Populus alba* (L.) (white poplar), and *Eucalyptus* spp*.* (no specific author for the genus), as well as *Platanus x hispanica* (Müller) (London plane) (Fig. 1).

### Study population

A total of 138 volunteers were screened for hypersensitivity to aeroallergens between 22 and 26 July 2024. Participants aged 18 years and above living near or on the Vaal Campus of NWU in Vanderbijlpark were involved in the study. The participants were included irrespective if they have known allergenic symptoms (i.e., skin itching, runny nose, asthma for at least 3 days or nights a week for a minimum of 3 months). Participation was voluntary, and the patients were given an “information sheet” to read and a “consent form” to complete and to provide consent for their participation. Patients were excluded from the study if they experienced uncontrolled asthma, poor lung function, recent anaphylaxis (during the previous 30 days), and skin conditions such as dermatographism, urticaria, mastocytosis, and atopic dermatitis (Bousquet et al., 2012). Further exclusion criteria include taking anti-allergy medication such as antihistamines at least 1 week before the SPTt (Bousquest et al. 2012).

### Symptoms questionnaire and SPT

The study was approved by the Health Sciences Research Ethics Committee (HREC) of NWU, South Africa (NWU-00061–24-S1). Research access to the NWU Vanderbijlpark campus was provided by the NWU Research Data Gatekeeper Committee (RDGC). The symptoms questionnaire was adapted from the International Study of Asthma and Allergies in Childhood (ISAAC) (Asher et al., 1995). SPT was performed according to international standards, with sensitization being defined as a positive reaction to SPT. Nineteen commercially available allergen extracts were used for all patients in alignment with common pollen and fungal allergens reported from southern African spore traps (Esterhuizen et al., 2023; Gharbi et al., 2023). Furthermore, the selection of our allergen testing panel was based on the recommendation testing aeroallergen panel of the South African allergic working groups (Richards et al., 2023). Pollen and fungal extracts were purchased from Immunotek (Spain), including allergens from Poaceae: *Cynodon dactylon* ((L.) Pers.) (Bermuda grass), *Lolium perenne* (L.) (ryegrass), *Zea mays* (L.) (maize), six mix grass: *Poa pratensis* (L.) (Kentucky bluegrass), *Festuca pratensis* (L.) (meadow fescue), *Dactylis glomerata* (L.) (orchard), *Phleum pratense* (L.) (Timothy grass), *Holcus lanatus* (L.) (Yorkshire fog), Cupressaceae: *Hesperocyparis arizona* (Greene) (Arizona cypress), Fagaceae: *Quercus robur* (L.) (English oak), Platanaceae: *Platanus sp.* (L.) (plane tree), Ulmaceae: *Ulmus campestris* (L.) (field elm), Betulaceae: *Betula verrucosa* (L.) (silver birch), Oleaceae: *Olea* (L.) (olive tree) sp., weed mix (*Artemisia sp.* (L.) (wormwood), *Chenopodium sp.* (L.) (goosefoot), *Salsola sp.* (L.) (saltwort), *Plantago sp.* (L.) (plantain)), Asteraceae: *Ambrosia* (L.) (ragweed) sp., fungal spores including *Aspergillus fumigatus* (L.), *Alternaria alternata* (Fr.), *Cladosporium* (Link) sp. and *Penicillium notatum* (Westend.). Due to unavailability from our supplier, *Morus* (L.) (mulberry) pollen was not included in the current study although it is highly allergenic, and the taxon is abundant in aerobiological monitoring (Neumann et al., 2024). Myrtaceae pollen has shown a moderate presence in the aerobiological records of nearby cities within the same vegetation and climate zone (e.g., Potchefstroom (Neumann et al., 2024), Johannesburg, Pretoria (Esterhuizen et al., 2023, Ajikah et al. 2023)), and was therefore excluded from consideration. Notably, Johannesburg is located 71 km away, and Potchefstroom is 77 km, with all cities exhibiting temperate grassland vegetation with nearby savanna patches. In addition to *Eucalyptus* (L’Hér.) (eucalyptus) spp. pollen, several other Myrtaceae taxa, such as *Callistemon* (L.) (bottlebrush), were recorded. However, these taxa are not included in commercially available allergy panels. Similarly, Combretaceae (L.) (indigenous bush willow) are not present in allergy panels. Other indigenous regionally occurring tree families such as *Senegalia* (Benth.) (acacias) spp. and *Vachellia* (Seem.) (acacias) spp. (Coates Palgrave, 2002) are infrequently recorded in South African aerobiological studies (see Esterhuizen et al., 2023; Neumann et al., 2024), with many not being represented in allergy panels. The current selection of fungal spores in the allergy test panels is based on those—similar to pollen types—recognized in Potchefstroom and Johannesburg in a similar biome/climate (most dominant in Potchefstroom are *Alternaria* (Fr.), *Cladosporium* (Link), see Neumann et al., 2024). SPTs were performed by two medical professionals (co-authors in this publication) with expertise in emergency management of anaphylaxis and other allergic reactions. SPT was performed on the forearm with a standardized solution of allergens. A mean wheal diameter of 3 mm or greater was taken to indicate the presence of specific IgE in the allergen tested.

### Statistical analysis

Code data were entered into a Microsoft Excel spreadsheet and analyzed using means with a range for normally distributed data and frequency (number and percentage) of participants as appropriate.

A Chi-Square test (*χ*^2^) was performed to determine the association between SPT reactions, gender, ethnicity, and smoking. Tests were performed using SPSS (IBM Corp, Version 29.0.2.0, released 2023, Armonk, NY). Ethnicity was grouped into two categories, “African” and “Other,” with “White”, “Asian,” and “Colored” grouped into “Other”. Age did not contain a distinct enough age range to be considered. Two significance levels were considered, 5% and 10%. That is, p-values less than 0.05 showed a statistically significant association, while *p*-values less than 0.1 were still considered significant, albeit with a weaker association.

## Results

### Demographic variables of participants

A total of 138 patients (51 males and 87 females) with a mean (range) age of 22 (18–56) years were involved in this study. Participants with African ethnicity comprised a total of 122 (88.4%) of the study population, and the remaining were mainly 7.2% colored, 1.4% white, 1.5% Asian, and 1.5% not specified.

### Detection of skin allergic sensitization to aeroallergens

After responding to the pre-screening ISAAC questionnaire, twenty-eight participants were excluded from the study due to histamine treatment or without any clear allergic symptoms after the first symptoms questionnaire pre-screening. Four participants had to be excluded due to invalid SPT results (wheal diameter < 3 mm). A total of 106 participants were clinically diagnosed with pollen and fungal spore allergy. Table 1 shows the demographic characteristics of participants with positive SPT. The gender distribution was 64.15% (68) women, 33.0% (35) men, and 2.83% (3) with no specified sex. The age group (18–23) recorded the highest positivity (82.7%), while the lowest skin prick test reaction was attributed to the age group above 35 (1.81%). Among patients with positive skin prick tests, 42.5% (45) reported having sneezing and running noses during the past 12 months, while 30.2% (32) confirmed the occurrence of itchy and watery eyes. Other symptoms reported by the participants included itchy rash 10.4% (11) and asthma 9.4% (10).

**Table 1:** The demographic variables of participants with positive SPT (*n* = 106)

Demographic variable	No	%
**Age**		
18–23	91	82.7
24–29	11	10.0
30–35	2	1.81
> 35	2	1.81
**Sex**		
Male	35	33.0
Female	68	64.15
Not specified	3	2.83
**Self-reported symptoms (ISAAC) during the past 12 months**		
**Allergic rhinitis**		
Sneezing, running, blocked nose out of flu or cold	45	42.5
Itchy watery eyes	32	30.2
Hay fever	8	7.5
**Eczema**		
Itchy rash	11	10.4
**Asthma**	10	9.4

**Fig. 2:**
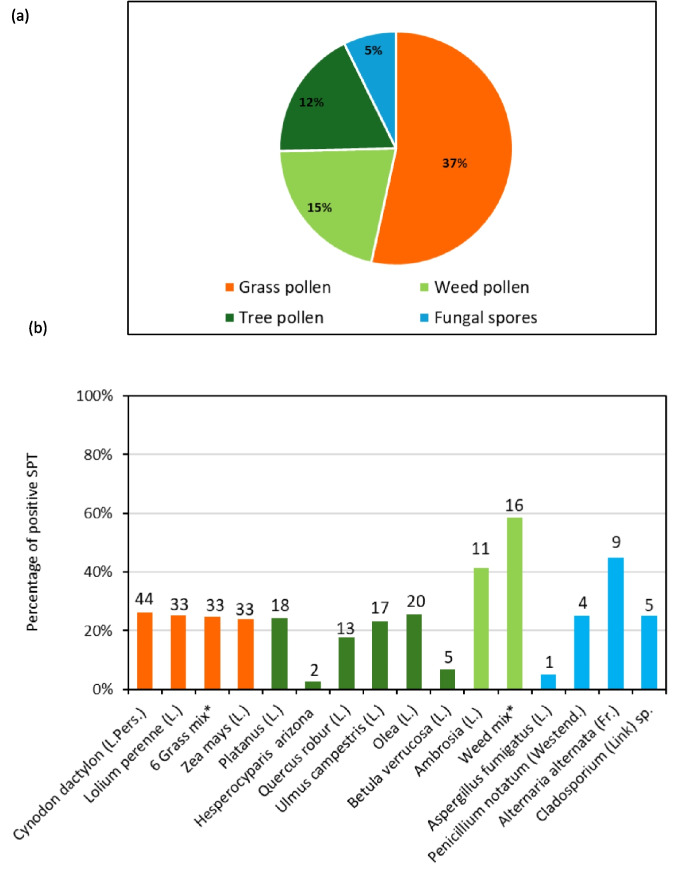
**a** Overall prevalence of aeroallergens categories; **b** Percentage pattern (frequency) of SPT-positive reactivity to specific pollen and fungi species among the studied population (*n* = 106). *6-grass mix: *Poa pratensis* (L.), *Festuca pratensis* (L.), *Dactylis glomerata* (L.), *Phleum pratense* (L.), and *Holcus lanatus* (L.); *weed mix: (*Artemisia* (L.), *Chenopodium* (L.), *Salsola* (L.), and *Plantago* (L.)

Figure 2a presents the overall prevalence of skin allergic sensitization positivity per category of trees, grass, and weed pollen, as well as fungal spores. The distribution of pollen hypersensitivity in the skin prick test showed that grass allergens were the most common outdoor allergens for all the examined participants. Detailed percentages of skin prick test positivity within the studied participants are presented in Fig. 2b. In total, 41.5% (44) presented positive skin allergic reactions to *Cynodon dactylon* (L.Pers.) (Bermuda grass), followed by 33 participants (31.1%) sensitized, respectively, to *Lolium perenne* (L.) (ryegrass), grass mix and *Zea mays* (L.) (maize). Moreover, among the tree allergens, *Olea* (L.) (olive) was the most prevalent allergen (20; 18.8%) followed by *Platanus* (L.) (plane tree) (18; 16.9%). Among the weeds, 16 (15.1%) participants were allergic to the weed mix (*Artemisia* (L.) (wormwood), *Chenopodium* (L.) (goosefoot), *Salsola* (L.) (saltwort), *Plantago* (L.) (plantain), and 11 (10.3%) to *Ambrosia* (L.) (ragweed). Regarding the fungal spores, *Alternaria* (Fr.) (9; 8.5%) followed by *Cladosporium* (Link) sp. (5; 4.7%) presented the highest skin sensitivity.

**Fig. 3:**
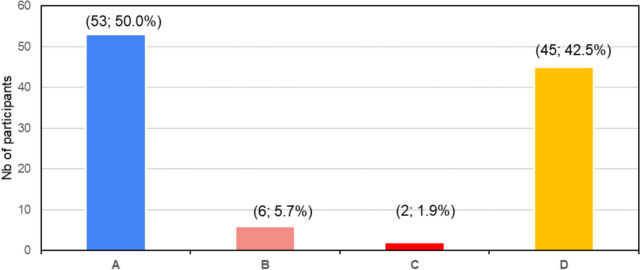
Frequencies of sensitization with SPT (in percentages), (**A**) monosensitization; (**B**) SPT positive to two allergens; (**C**) SPT positive to three allergens; (**D**) SPT positive to four or more allergens

Figure 3 shows the frequency of sensitization among the 106 participants. Out of all the participants, 50% (53) were monosensitized and showed a clear positive reaction to one allergen, while 50% (53) were polysensitized to more than two or three pollen types or fungi. A total of 45 participants (c. 43%) showed positive reactions to four or more allergens.

### Allergen sensitization across gender, ethnicity, and smoking status

The results of aeroallergen testing between gender groups showed that the positivity rate of *Ambrosia* pollen allergen was considerably higher in females than in males (13.9% vs. 2.9%, *χ*^2^ = 3.10, *p* = 0.098) (Table 2). No statistically significant association was found in the positivity rate of the other pollen types between the two gender groups (*p* > 0.05) (Table 2). We analyzed the pollen allergen positivity rates with regard to participants’ ethnicity and found a significant association only in the *Platanus* (Plane Tree) and *Cynodon dactylon* (L. Pers.) allergen positivity rates between the African and Non-African groups (Colored, Asian, White) (Table 2).

**Table 2:** Chi-square analysis of allergen sensitization across gender, ethnicity, and smoking status, with corresponding percentages and *p*-values

	**Gender**				**Ethnicity**				**Smoking**			
Allergen	***χ***^**2**^	***p***	**% F**	**% M**	***χ***^**2**^	***p***	**% AF**	**% Oth**	***χ***^**2**^	***p***	**% N-SM**	**% SM**
*Aspergillus fumigatus*	0.491	1.000	1.4%	0.0%	0.13	1.000	1.1%	0.0%	0.218	1.000	1.1%	0.0%
*Penicillium notatum*	0.564	0.596	2.8%	5.7%	0.757	0.389	3.2%	8.3%	0.897	1.000	4.5%	0.0%
*Alternaria alternata*	2.083	0.266	11.1%	2.9%	1.27	0.593	9.7%	0.0%	0.134	0.659	8.0%	10.5%
*Cladosporium* sp.	0.385	1.000	5.6%	2.9%	0.677	1.000	5.4%	0.0%	1.132	0.583	5.7%	0.0%
*Cynodon dactylon* (Bermuda grass)	0.012	1.000	38.9%	40.0%	4.342	0.050*	35.5%	66.7%	1.734	0.205	36.4%	52.6%
*Lolium perenne* (ryegrass)	0.057	0.825	31.4%	29.2%	0.96	0.331	28.0%	41.7%	3.36	0.096**	26.1%	47.4%
6-grass mix*1	0.057	0.825	31.4%	29.2%	0.133	1.000	30.1%	25.0%	0.53	0.581	28.4%	36.8%
*Platanus* sp. (plane tree)	0.004	1.000	16.7%	17.1%	5.737	0.031*	14.0%	41.7%	3.595	0.086**	13.6%	31.6%
*Hesperocyparis arizona* (Arizona cypress)	4.193	0.105	0.0%	5.7%	2.997	0.216	1.1%	8.3%	0.44	1.000	2.3%	0.0%
*Quercus robur* (English oak)	2.018	0.214	15.3%	5.7%	0.229	0.642	11.8%	16.7%	8.171	0.011*	8.0%	31.6%
*Ambrosia* sp. (ragweed)	3.107	0.098**	13.9%	2.9%	0.066	1.000	10.8%	8.3%	2.907	0.104	8.0%	21.1%
*Zea mays* (maize)	0.436	0.653	31.9%	25.7%	0.094	0.745	29.0%	33.3%	0.53	0.581	28.4%	36.8%
*Ulmus campestris* (field elm)	0.679	0.573	18.1%	11.8%	0.001	1.000	16.3%	16.7%	0.616	0.482	14.8%	22.2%
*Olea* sp. (olive tree)	0.566	0.597	20.8%	14.7%	1.737	0.239	17.4%	33.3%	1.124	0.325	17.0%	27.8%
*Betula verrucosa* (silver birch)	2.55	0.170	6.9%	0.0%	0.381	0.462	4.3%	8.3%	1.777	0.215	3.4%	10.5%
Weed mix*2	0.508	0.573	16.7%	11.4%	0.392	1.000	15.1%	8.3%	0.013	1.000	14.8%	15.8%

Author names for each taxon are available in the text but not provided here for brevity

*F* female, *M* male, *AF* African, *Oth* others (Asian, White, Colored), *N-SM* non-smoker, *SM* smoker

*1 *Poa pratensis* (Kentucky bluegrass), *Festuca pratensis* (meadow fescue), *Dactylis glomerata* (orchard), *Phleum pratense* (Timothy grass), *Holcus lanatus* (Yorkshire fog)

*2 *Artemisia* (wormwood), *Chenopodium* sp. (goosefoot), *Salsola* sp. (saltwort), *Plantago* sp. (plantain)

*p-value < *0.05*; **p-value < *0.1*

Considering the association between smoking factor and allergen exposure, a significant association was observed only in Quercus L. (oak), *Lolium perenne* (L.) (ryegrass), and *Platanus* (L.) (Plane Tree). The remaining aeroallergen did not differ from the smoking factors (Table 2).

## Discussion

The skin prick reactivity indicated a range of allergic responses against pollen and fungal spore allergens among the participants (see Fig. 2a and b). The prevalence of SPT reactivity to pollen in this study range was 64%, whereas fungal spores were comparably minor with a prevalence of 5%.

Grass pollen exhibits a prevalence of 37% SPT reactivity. Not surprisingly, pollen of Poaceae—highly allergenic and abundant in grass-dominated environments such as the Grassland and Savanna biomes (Esterhuizen et al., 2023) —was triggering most of the positive SPT reactions during the current study. The Poaceae taxon that showed the highest degree of positive reaction (total, 44) is *Cynodon dactylon* ((L.) Pers.) (Bermuda grass) which is indigenous to South Africa (Roodt et al., 2002). This is in good agreement with a previous study analyzing AMPATH datasets from South Africa where *Cynodon dactylon* ((L.) Pers.) (Bermuda grass) and *Phleum pratense* (L.) (Timothy grass) were the most prevalent aeroallergens (Murray et al., 2022). *Phleum pratense* (L.) (Timothy grass) was in the current study only tested as a component of the 6-grass mix (see Fig. 2b). *Lolium perenne* (L.) (exotic ryegrass), recorded as an herbicide-resistant weed in the Western Cape (Ferreira et al., 2015), shows high reactivity in the SPT (total: 33).

Amongst the tree pollen, *Olea* (L.) (olive), *Platanus* (L.) (plane tree), *Ulmus* (L.) (elm), and *Quercus* (L.) (oak)- all originating from the northern hemisphere except for Olea which includes both indigenous and exotic taxa- were causing most of the allergic reactions in alignment with highly abundant pollen of those taxa in spore traps in Gauteng (Pretoria, Johannesburg) (Esterhuizen et al., 2023). *Olea europaea* L. ssp. *africana* ((Mill.) P.S.Green) (wild olive), *Olea capensis* (L.) (ironwood), and *Olea exasperata* (Jacq.) (sand olive) are indigenous to South Africa (Coates Palgrave, 2002). However, the latter two taxa are restricted to the Cape, although *O. capensis* also grows in some other SA provinces but not in North West (Coates Palgrave, 2002). The European olive, *Olea europaea* (L.) (common olive), is planted as a fruit tree at the Cape and as an ornamental tree throughout the subcontinent (Cariñanos & Marinangeli, 2021; Neumann et al., 2024).

In contrast, pollen of other highly dispersed and allergenic neophytic trees such as *Hesperocyparis* (Bartel & R.A.Price) (cypresses) sp. (Esterhuizen et al., 2023) rarely triggered positive reactions although cypresses are common in the region (compare Neumann et al., 2024).

The study revealed that the weed mix and *Ambrosia* (L.) (ragweeds) (total of 27 positive reactions) showed high reactivity during the SPT. *Ambrosia* (L.) (ragweeds) spp*.*, North American weeds which are currently spreading in South Africa, deserve special attention due to the high allergenicity of the pollen (Chen et al., 2018, Gharbi et al., 2024).

Allergic sensitization patterns differ globally; a rise in sensitization to pollen in children and adults with respiratory allergy was documented in a very comprehensive study in Spain (Ojeda et al., 2018). Ojeda et al. (2018) pointed out an increase prevalence of pollen by 50% between 2005 and 2015, with percentages relevant of 73.7% in grasses, 52.1% in *Olea europea* (L.) (olive), and 22.8% in *Cupressus* species being the most common allergens during 2015. More recently, a study conducted in Mexico (Larenas-Linnemann et al., 2024) aimed to compare the sensitization patterns between 2009 and 2023 and demonstrated an increase in SPT positivity for almost all the most frequent allergens such as *Cynodon dactylon* 27% (2023) vs 23% (2009), *Lolium perenne* 24% (2023) vs 14% (2009), and *Quercus* 23% (2023) vs 19% (2009). Data reported from Australia, a Southern Hemisphere country, are in agreement with our findings. A high frequency of SPT was reported for *Cynodon dactylon* 84% (Davies et al., 2012).

Multiple positive reactions in SPTs, as shown in the current study, where a majority of positively tested participants showed a reaction against four or more allergens (see Fig. 3), may indicate cross-reactivity between structurally similar allergens, polysensitization to diverse allergens, environmental exposure, or a genetic atopic predisposition (Migueres et al., 2014). Generally, more than 50% of patients seeking consultation or testing for respiratory allergies are polysensitized (Migueres et al., 2014). In the current study, we report a polysensitization for c. 42.5% of all participants (positive to four or more allergens) whereas for c. 50% of the participants’ monosensitization is documented (see Fig. 3). This might point to moderate exposure to aeroallergens, which needs to be confirmed by future aerobiological studies. The potentially high degree of cross-reactivities with, e.g., food allergens amongst the participants (compare Murray et al., 40) will motivate to conduct further SPTs with a focus on other allergens in addition to aeroallergens. Polysensitizations can complicate diagnosis and exacerbate symptoms which highlights the need for a careful management of allergic conditions (Migueres et al., 2014). It is also important to consider that in a highly air-polluted region like VTAPA, where Vanderbijlpark is located, the prevalence of respiratory diseases, including severe asthma, is already high and will continue to rise (Oostuizen et al. 2014). This stands in contrast to European studies with similar asthma increases from 1990 to 2020, where the percentage of asthmatic children on medication was decreasing (Vermeire et al., 2002).

The results of this study emphasize the importance of allergy screening and diagnosis in South Africa, especially due to the evidenced abundance of aeroallergens from exotic trees and weeds, which might further spread under conditions of climate change (Van Wilgen et al., 2020, see Esterhuizen et al., 2023, Neumann et al., 2024). In the current study, by combining the ISAAC questionnaire with SPTs, individuals with allergic sensitizations were successfully identified, marking a critical step in managing allergic diseases.

The ISAAC questionnaires screen not only for airborne pollen grains and fungal spores present in the atmosphere but also for allergic rhinitis caused by various inhalants, such as animal dander and house dust mites. Although this study shows that participants have aeroallergens sensitivity, it is also important to mention that another group of participants with AR symptoms and negative SPT could be explained by local allergic rhinitis (Rondón et al., 2012).

Our results showed only a significantly higher positive rate for *Ambrosia* pollen, among females compared to male participants (Table 2). This supports previous findings that females have a higher allergy prevalence due to the mechanistic involvement of sex hormones in immune reactions (Jensen-Jarolim and Untersmayr 2008). *Platanus* (L.) (plane tree) was significantly associated with ethnicity (African) at *α* = 0.05 (Table 2). This is in good agreement with previous studies, where Wegienka et al. (2013) postulate that blacks are more frequently sensitized than white individuals. However, data on other racial groups is limited. Genetics are unlikely to be the primary cause of these differences, and home dust allergen and endotoxin levels do not account for them (Wegienka et al., 2013). *Cynodon dactylon* (Bermuda grass) showed a trend at *p* = 0.1, with the “Other” ethnicity group exhibiting the most positive reactions (Table 2). This emphasizes that further research is needed to identify the sources of racial disparities (Wegienka et al., 2013). Smoking status was significantly associated with a positive reaction to *Quercus* (L.) (oak) at *p* = 0.05 (Table 2). *Lolium perenne* (ryegrass) and *Platanus* (L.) (plane tree) showed trends at *p* = 0.1, with smokers presenting higher reaction rates (Table 2). This seemingly supports studies that evidenced a significantly higher prevalence of allergic rhinitis in current and passive smokers compared to nonsmokers (Mlinaric et al., 2011). However, conflicting evidence exists (compare Grillo et al., 2019), and a contrasting study postulates that smokers may exhibit lower allergy expression compared to non-smokers (Mishra et al., 2008).

## Conclusions and outlook

This study presents findings from initial research that indicate a significant prevalence of pollen allergies. Next to grasses (both native and introduced taxa), predominantly pollen of northern hemisphere exotic trees such as *Platanus* (L.) (plane tree) and *Quercus* (L.) (oak) next to pollen of partly indigenous *Olea* (L.) (olive tree) were triggering positive SPT reactions. We recommend considering taxon-specific pollen allergy risks when planning urban tree cultivation. Amongst the weeds, *Ambrosia* (L.) (ragweed) spp. needs to be closely monitored due to its high allergenicity and current spread in South Africa. The South African novel approach of combining ISAAC screening with skin prick testing provides a robust method for allergy diagnosis.

Limitations of the current study area are as follows:

a Unbalanced age range since the study, was conducted on a university campus, which might cause a bias when evaluating allergic reactions for the whole population of the region;
b The SPT results presented here are gathered on a university campus and are therefore not representative of the total population of the area which will be addressed in a 2025 SPT in the township of Sharpeville with a more mixed socioeconomic background where also a more balanced age range and ethnic diversity can be expected;
c Number of participants is comparably low but will be enhanced by further SPTs planned for 2025;
d A further limitation of the current study is that, with highly allergenic Morus pollen, an important allergen was not tested, further allergens—if available—can be added to the pollen allergy test panel if additional important allergens are discovered in future local aerobiological studies. Ongoing pollen monitoring in VTAPA, at Vanderbijlpark and Sharpeville, will help to understand the linkage between pollen allergy sensitivities in the local population and the abundance of specific allergenic pollen and fungal spores in the regional atmosphere;
e Future research should focus on a better understanding of interlinkages between air pollution levels and (pollen) allergies. This would underline a focus on highly air-polluted regions.

In general, further research is needed to extend these findings to broader populations in other South African provinces and to develop effective public health strategies to address the rising burden of allergic diseases in South Africa. The study’s findings can inform public health interventions spearheaded by the South African Medical Research Council (SAMRC), aimed at reducing the burden of allergies in South Africa. The research strategy for understanding pollen allergies in South Africa could involve collaboration with other universities. especially University of Cape Town and University of Pretoria which both have a current focus on allergy and air pollution research, the CSIR, and government health agencies to provide scientific expertise and data analysis. Engaging with public health organizations and raising awareness would help translate findings into effective interventions.

## Data Availability

No datasets were generated or analysed during the current study.
